# Special Number or a Mere Numerical Array? Effect of Repdigits on Judgments and Choices

**DOI:** 10.3389/fpsyg.2020.01551

**Published:** 2020-07-16

**Authors:** Hidehito Honda, Sota Matsunaga, Kazuhiro Ueda

**Affiliations:** Graduate School of Arts and Sciences, The University of Tokyo, Tokyo, Japan

**Keywords:** effect of numerical arrays, repdigits, rarity of numerical arrays, preference of number, randomness of number

## Abstract

Previous studies have shown that people find special meaning in numerical arrays. In this article, we have focused on the features of numerical arrays, repdigits (e.g., “777”), and examined the effect of repdigits on judgments and choices. We formulated the following hypotheses: (1) when people want to assign special meanings to numbers [in the case of purchase or choice of alternatives that contain numbers (e.g., serial numbers)], repdigits will be chosen since people tend to prefer numbers that contain repdigits, and (2) when people think about probabilistic or statistical events involving numerical arrays, they will regard repdigits as a mere set of numerical arrays, and preference for them will disappear. Through five behavioral experiments, we examined these two hypotheses and the results generally supported them. We also discussed the features and psychological processes of repdigits in judgments and choices.

## Introduction

Previous studies revealed that people are often affected by superficial factors that are essentially unimportant in various decisions and judgments. For example, target representation such as font readability, linguistic script, and abstract dots versus actual pictures affect people’s decisions, judgments, categorization, and image generation (Hsee and Rottenstreich, 2004; Alter and Oppenheimer, 2008a, b; Oppenheimer and Frank, 2008; Song and Schwarz, 2008a, b; Pocheptsova et al., 2010; Honda et al., 2018). This indicates that people tend to find meaning in superficially inessential features of targets, and this may hold true for the various numerical arrays that people see in their daily lives. In addition to essential meanings, they may sometimes find special meanings in numerical arrays.

Various studies have discussed the effects of numerical arrays on people’s psychological processes; people have unique preferences for lotteries (Farrell et al., 2000), date of birth (Kitayama and Karasawa, 1997), and round numbers (Pope and Simonsohn, 2011; Allen et al., 2017). These results indicate that people find special meanings in numerical arrays and show unique preferences or considerations.

In the present study, we investigated the effect of repdigits on people’s judgment and choices. Repdigits denote numerical arrays containing numbers with the same digit repeated, such as “777” or “555.” They are sometimes associated with “special” meanings. For example, “777” represents the jackpot of a slot machine, and “666” represents the “Number of the Beast” in the Book of Revelation. In addition, the economic research of Kabátek and Ribar (2018) showed that in Netherlands, the incidence of weddings was higher on numerically special days (dates of sequential number values, e.g., 9.9.99). Repdigits are actually highly “special” numerical arrays since they are rare. The proportion of repdigits in three-digit numbers is 10/1000 (i.e., “000,” “111,”…, “888,” and “999”), while in four-digit numbers, it is 10/10000 (i.e., “0000,” “1111,”…, “8888,” and “9999”). Rarity affects our psychological processes such as hypothesis testing (McKenzie and Mikkelsen, 2000), covariation assessment (McKenzie and Mikkelsen, 2007), probability judgments (Dai et al., 2008), or frame choice (Honda and Matsuka, 2014). Since people tend to be sensitive to such rarities, repdigits may affect their psychological processes.

In the present study, we formulated the following hypothesis (rarity hypothesis) about the effect of repdigits on choices and judgments. We hypothesized that people actually pay attention to dichotomization between repdigits and non-repdigits and perceive a “rarity” in repdigits (i.e., they accurately discriminate between the two classes in terms of actual frequency of possible numerical arrays). Furthermore, such a perception would affect their judgments and choices. For example, repdigits may be preferred since such numbers are easily memorable (Hunt, 2006). As another example, given that a thing becomes more valuable in psychological and economic sense as it becomes rarer (Cialdini, 2001; Hirshleifer et al., 2006), people may find repdigits “valuable.” Based on these considerations, we made the following specific predictions about the effects of repdigits.

*Prediction 1*: Repdigits will be preferred when people want to assign special meanings to numbers such as “easily memorable” or “valuable.”

For example, imagine that for your party, you are planning a game of dice, with red and blue dice. For some outcomes of rolling the dice, the players of the game could win a prize. Which outcome will you choose for winning? In this case, you may prefer repdigits such as “red 1, blue 1,” or “red 6, blue 6,” since you may want to assign “easy” numbers to memorize or add special values for numbers.

However, repdigits may not always affect our judgments or choices. Imagine that in rolling the aforementioned red and blue dice, you are asked, “Which is more probable as the outcome of rolling the two dice: red 1, blue 1 or red 3, blue 4?” The two outcomes are equally probable (1/36). In such a probabilistic or statistical question, people may not necessarily pay attention to the difference in rarity between repdigits and non-repdigits but regard each numerical array just as one of the sets of a numerical array. Thus, we made the following prediction.

*Prediction 2*: When people think about probabilistic or statistical events wherein numerical arrays are involved, people do not pay attention to the difference between repdigits and non-repdigits. In this case, repdigits will not affect their judgments or choices, and preference for them will disappear.

We note that the target questions in Predictions 1 and 2 are essentially different from each other. In Prediction 1, the target question is about people’s subjective preference. Contrarily, in Prediction 2, the target question is about objective value. Therefore, we predict that repdigits will only affect our choices or judgments regarding subjective preferences.

In the present study, we conducted five behavioral experiments in total, to examine the above two predictions.

## Experiment 1

### Method

#### Participants

The participants included 296 Japanese women and 304 men (*N* = 600), with a *M*_age_ = 44.38 and SD_age_ = 8.18. All participants were recruited via a website and received coupons that could be redeemed for online shopping in Japan. They had to answer two out of the four questions.

To the best of our knowledge, this was the first experimental study to examine the effect of repdigits on judgments and choices. Therefore, we took a conservative stand on this task’s effect size. When we set the small effect of *h* = 0.2 (Cohen, 1988), and 90–95% power with the alpha level of 0.05, we needed around 260–320 participants. Based on this analysis, we set the number of participants at 300 for each question.

#### Task, Materials, and Procedure

The participants answered the binary-choice question about a birthday. They were presented with the name of a target and two alternatives. One of the questions was “When is Bob Dylan’s birthday, 2/19 or 2/22?”^1^. The participants were asked to choose one of the birth dates for the target based on what they thought was the correct answer.

Four questions (see Table 1) constructed from four target names and two sets of alternatives were provided. The present task was conducted in one of the serial tasks in a web-based experiment while the other tasks were irrelevant to it. Participants answered two of the four questions [one of (2–1 or 2–2), and one of (8–1 or 8–2)], and each participant answered one “hypothetical” and one “real” target.

**TABLE 1:** Targets and questions in Experiment 1.

Question ID	*n*	Target	Target type	Question
2-1	300	Bob Dylan	Real	When is his birthday, 2/19 or 2/22?
2-2	300	Kumamon	Hypothetical	
8-1	300	Donald Trump	Real	When is his birthday, 8/8 or 8/17?
8-2	300	Suneo	Hypothetical	

*“Kumamon” is a mascot created by the Government of Kumamoto prefecture, while “Suneo” is a manga character in the Doraemon series.*

For each target, the real birthday is either a different day or unclear. The goal of the present task was to examine whether participants thought that repdigit birthdays were probable. We did not focus on whether participants actually knew the target’s birthday; we randomly chose the alternatives. After the task, participants were asked whether they knew the target’s birthday before this experiment, and none of them had known it. We also confirmed that all the participants recognized the four targets.

### Results and Discussion

Our focus was on clarifying whether the choice pattern would change depending on the target type. Based on Predictions 1 and 2, for the “hypothetical” (the targets are not actual humans or animals, such as a manga character or mascot) target, participants would choose the repdigit birth date (i.e., 2/22 or 8/8) since they would find it easy to memorize as a unique character or attribute special meaning for the birthday. Thus, participants would find the repdigit birth date appropriate for the hypothetical target’s birthday. In contrast, for the “real” target, the task was related to the statistical judgment of actual birth date. Participants would regard each birthday in the two alternatives as one of the days in a year and equally possible as a birthday. Since participants did not know the target’s birthday, their choice would be made based on chance (i.e., 50%).

**FIGURE 1:**
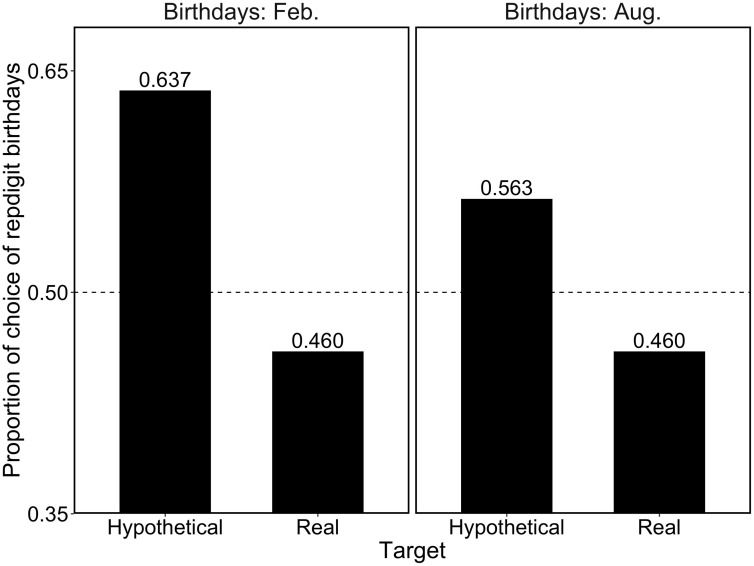
Proportion of repdigit birthdays as a function of the target type. The dotted line (0.5) indicates the random choice.

Figure 1 shows the proportions of repdigit birthdays as a function of target type. First, we compared the proportions between the target types. For the question about the birthday in February, participants in the hypothetical target condition chose the repdigit more than those in the real target condition [χ^2^(1) = 18.20, *p* < 0.001, *h* = 0.36]. Likewise, for the question about birthdays in August, participants in the hypothetical target condition chose the repdigit more than those in the real target condition [χ^2^(1) = 6.00, *p* < 0.01, *h* = 0.21], consistent with our prediction.

Next, we examined whether the choice rate of repdigit birthdays deviated from the random choice pattern (i.e., 50%) using a binomial test. It was found that for the hypothetical target, participants significantly chose the repdigit birthday (February birthday, *p* < 0.001; August birthday, *p* = 0.03). Contrarily, significant deviations from the random choice pattern was not observed in the real target (February birthday, *p* = 0.18; August birthday, *p* = 0.18). These results corroborated our hypotheses.

## Experiment 2

In Experiment 1, we found that when participants answered the questions about the real target’s birthday, they did not show a significant preference for repdigits. We tested whether this finding could be observed in a different (and more direct) context.

### Method

#### Participants

The participants were 148 women and 152 men (*N* = 300) with a *M*_age_ = 44.52, SD_age_ = 8.30. All participants were recruited via a website and received coupons that could be redeemed for online shopping in Japan.

Since we examined the null hypothesis in Experiment 2, we adopted a conservative position on the effect size for this task. As in Experiment 1, for each question, we set the number of participants as 300.

#### Task, Materials, and Procedure

The participants were first presented with the following instructions:

Here is a gamble with 10 alternatives, from which, one will be randomly picked up as “winning.” Which do you imagine is most probable to be picked up as “winning”?

The participants were then presented with a set of alternatives as in Table 2 (i.e., G1 or G2) and were asked to choose one of the alternatives for each set.

**TABLE 2:** Presented alternatives in Experiment 2.

Question ID	Presented alternatives
G1	110, 111, 112, 113, 114, 115, 116, 117, 118, 119
G2	220, 221, 222, 223, 224, 225, 226, 227, 228, 229

The present task was conducted in one of the serial tasks in a web-based experiment, while the other tasks were irrelevant to it. Participants answered both the questions, G1 and G2. The order of presentation for the two sets was randomized for each participant, and irrelevant tasks were inserted between the two sets.

### Results and Discussion

**FIGURE 2:**
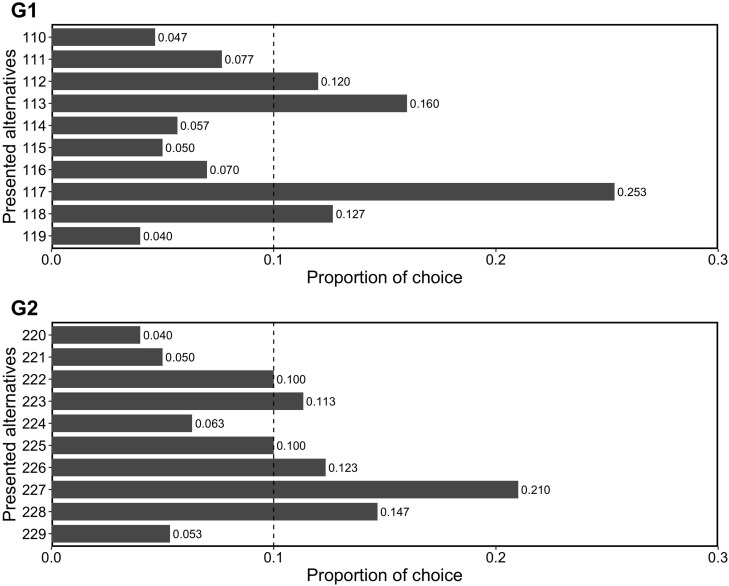
Proportion of choice in Experiment 2. The dotted line (0.1) indicates the random choice.

We examined whether repdigit alternatives (i.e., 111 in G1 and 222 in G2) were significantly chosen and predicted that this pattern would not be observed. Figure 2 shows the results of choices. We analyzed whether the repdigit alternatives were significantly selected as compared to random choice proportion (i.e., 0.1) using a binomial test. We found that repdigits were not significantly chosen (in G1, *p* = 0.210; in G2, *p* = 1). Thus, our prediction was corroborated.

On the whole, participants preferred the alternative of 7 in G1 and G2. A general preference for 7, known as “Blue Seven Phenomenon” (Simon, 1971; Simon and Primavera, 1972; Trueman, 1979; Vandewiele et al., 1986; Silver et al., 1988; Saito, 1999), was replicated in the present study, too. Japanese people prefer the number 7 as it is considered “a lucky number” and “represents happiness” (Saito, 1999). However, beyond this preference, participants did not show a preference for repdigits, as we predicted.

## Experiment 3-A

Two issues were analyzed: first, we examined the findings of Experiment 1 on preference for repdigits in different domains by using a wine bottle and a car license plate as the choice task. Second, we investigated whether there were individual differences in choice. For example, in choosing a wine bottle, those who have a strong interest in drinking wine, collecting wine bottles, or are knowledgeable about wine, may be particular about the bottle number, whereas to the rest, the bottle numbers may not matter. We examined whether individual interests on a target affected the preference for repdigits.

### Method

#### Participants

A total of 484 participants were recruited via a website. These included 241 participants who had a special interest in wine but not in cars (101 women and 140 men; *M*_age_ = 46.86, SD_age_ = 8.85, hereafter referred to as “Wine-interest group”), and 243, who had a special interest in cars but not in wine (16 women and 227 men; *M*_age_ = 46.45, SD_age_ = 7.50, hereafter referred to as “Car-interest group”). The operational definitions of “Wine-interest” and “Car-interest” groups were “spending more than 10,000 yen (approximately $100) on purchasing bottles of wine every month” and “purchasing at least one car-related magazine every month,” respectively. For their participation, they received coupons that could be redeemed for online shopping in Japan.

In Experiment 1, the effect size of repdigits ranged from *h* = 0.21 to *h* = 0.36. When we set the effect size at *h* = 0.3, and 90–95% power with the alpha level of 0.05, we needed around 115–140 participants. Based on this analysis, we recruited around 120 participants for each question and group.

#### Task, Materials, and Procedure

Participants answered two questions on their choice of wine bottle and car license plate. In the task pertaining to wine bottle choice, participants were presented with the following cover story:

You are supposed to buy a bottle of wine as a birthday present for your friend who is a wine lover. You decided to buy “Comte Georges de Vogue Bonnes-Mares 2014” (68,000 yen). You are now at a wine shop and there are four bottles. Which bottle will you choose?

Each participant was presented with either of the choice tasks: W1 or W2 (see Table 3).

**TABLE 3:** Choice tasks (wine bottle and car license plate) in Experiment 3-A.

Choice task about wine bottle		
Question ID	*n*	Presented bottle numbers
W1	120 (Wine-interest group) 125 (Car-interest group)	111, 112, 113, 114
W2	121 (Wine-interest group) 118 (Car-interest group)	221, 222, 223, 224

**Choice task about car license plate**		
**Question ID**	***n***	**Presented car license plates**

C1	120 (Wine-interest group) 122 (Car-interest group)	11-11, 11-12, 11-13, 11-14
C2	121 (Wine-interest group) 121 (Car-interest group)	22-21,22-22, 22-23, 22-24

In the task pertaining to car license plate choice, participants were presented with the following cover story:

You have bought a car, and out of the four car license plates, you can choose the one you like by paying 4,500 yen. Which license plate will you choose?

Each participant was presented with one of the choice task C1 or C2 (see Table 3).

Both tasks were conducted in one of the serial tasks as part of a web-based experiment while the other tasks were irrelevant to them. Participants answered two out of the four questions (one from W1 or W2, and one from C1 or C2). The order of presentation for the two questions was randomized for each participant, and irrelevant tasks were inserted between the two questions.

### Results and Discussion

**FIGURE 3:**
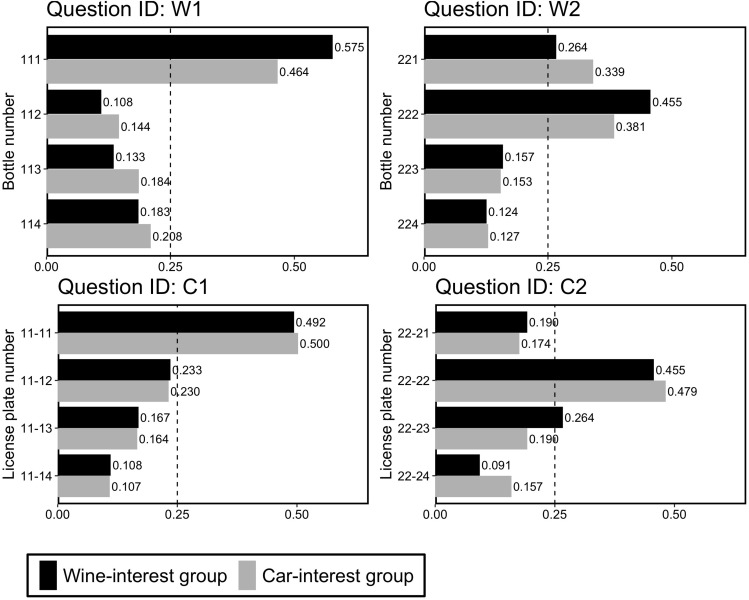
Proportion of choice in Experiment 3-A. The dotted line (0.25) indicates the random choice.

Figure 3 shows the proportions of choices for the four questions. First, on comparing the proportions between the groups, we found that there were no significant differences in choice patterns between the two interest groups for each question [Question W1, χ^2^(3) = 3.25, *p* = 0.35, *V* = 0.12; Question W2, χ^2^(3) = 1.88, *p* = 0.60, *V* = 0.09; Question C1, χ^2^(3) = 0.02, *p* = 1.00, *V* = 0.01; Question C2, χ^2^(3) = 3.78, *p* = 0.29, *V* = 0.12]. These results indicated that participants’ interests did not significantly affect their choices. Accordingly, in the following analyses, we merged the data between the two groups. Next, we examined whether participants preferred the repdigit wine bottle or license plate. Since we found that participants preferred the repdigit number in each question (see Figure 3), we examined whether this preference was a deviation from the random choice pattern (i.e., 25%) using a binomial test. We found that for all the four questions, participants significantly chose repdigits (*p* < 0.0001).

Taken together, the findings in Experiment 1 about repdigit preferences were also replicated in different domains. We also observed that participants tended to prefer repdigits regardless of their interests in the target.

## Experiment 3-B

Although the results of Experiment 3-A supported our hypothesis, some researchers may suspect that the results may have been obtained from the option set, which comprised one repdigit option and three non-repdigit options. Thus, repdigit option was rare in this option set, and it remains unclear whether “rarity of repdigit” or “rarity of option in the context” influences affected choices. In Experiment 3-B, we constructed the choice situation such that the choice set comprised three repdigit options and one non-repdigit option. That is, non-repdigit was “rare in this context.”

### Method

#### Participants

A total of 244 participants (93 women and 151 men; *M*_age_ = 48.30, SD_age_ = 12.40) were recruited via a website. The results in Experiment 3-A showed that each interest group (around 120 participants) showed a significant preference for repdigit in each question. According to this finding, we recruited around 120 participants for each question. Since the results in Experiment 3-A showed that special interests for wines or cars did not significantly affect choices, we did not control participants’ interests in this experiment.

#### Task, Materials, and Procedure

Except for option sets, task, materials, and procedure were basically the same as in Experiment 3-A. Table 4 shows the option sets used in Experiment 3-B. The options comprised three repdigit options and one non-repdigit option. Thus, as opposed to Experiment 3-A, the non-repdigit option was a rare option in the set.

**TABLE 4:** Choice tasks (wine bottle and car license plate) in Experiment 3-B.

Choice task about wine bottle		
Question ID	*n*	Presented bottle numbers
W3	129	222, 666, 999, 173
W4	115	222, 666, 999, 458

**Choice task about car license plate**		
**Question ID**	***n***	**Presented car license plates**

C3	117	22-22, 66-66, 99-99, 10-73
C4	127	22-22, 66-66, 99-99, 40-58

Two tasks were conducted in one of the serial tasks as part of a web-based experiment while the other tasks were irrelevant to them. Participants answered two out of the four questions (one from W3 or W4, and one from C3 or C4). The order of presentation for the two questions was randomized for each participant, and irrelevant tasks were inserted between the two questions.

### Results and Discussion

**FIGURE 4:**
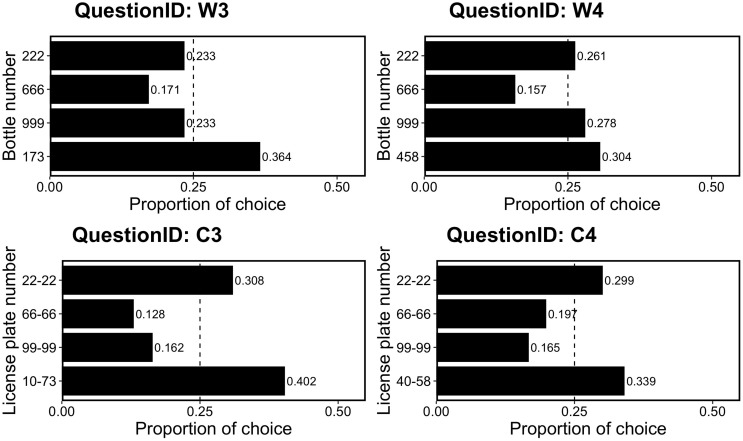
Proportion of choice in Experiment 3-B. The dotted line (0.25) indicates the random choice.

Figure 4 shows the proportions of choices for the four questions. We examined whether participants preferred the non-repdigit bottle or license plate, which was rare in the option set. As in Experiment 3-A, we examined whether this preference was a deviation from the random choice pattern (i.e., 25%) using a binomial test. We found that in the W3, C3, and C4 questions, participants significantly chose non-repdigit (W3, *p* = 0.004; C3, *p* = 0.0004; C4, *p* = 0.02), whereas in W4, significant preference for non-repdigit option was not observed (*p* = 0.20).

These results suggested that rarity in context affects people’s choice. Thus, the results of Experiment 3-A could have been explained through this perspective. For this examination, we compared the results from Experiment 3-A and those from Experiment 3-B using the Bayesian estimation method – as described by Kruschke (2014). The specific procedure is as follows: before Experiments 3A and 3B were conducted (since we could not make specific predictions about choice rates of rare options), we assumed a “vague” prior distribution using beta distribution. The beta distribution has two parameters, α and β. When *X* follows a beta distribution, its density *p*(*X*) is described as follows:

(1) $$p\,\left(X\right)=\frac{\Gamma \,\left(\alpha +\beta \right)}{\Gamma \,\left(\alpha \right)\,\Gamma \,\left(\beta \right)}\,X^{\alpha -1}\,{\left(1-X\right)}^{\beta -1}$$

where Γ(*z*) is the gamma function. For the “vague” prior, we set α and β as 1. Starting with this prior distribution, we computed the posterior distribution after the experiment with α = *1* + *z* and β = 1 + (*N – z*), where *N* indicates the whole number of participants and z indicates the number of participants who chose the rare option. We then estimated the choice rate based on a 95% highest density interval (HDI) for the posterior distribution. In this estimation, we merged the data along the line of question type (i.e., wine or car license plate) and experiment (i.e., Experiment 3A or 3B), respectively. That is, W1 and W2, C1 and C2, W3 and W4, and C3 and C4 were merged; we subsequently compared estimated choice rates for each question type.

**FIGURE 5:**
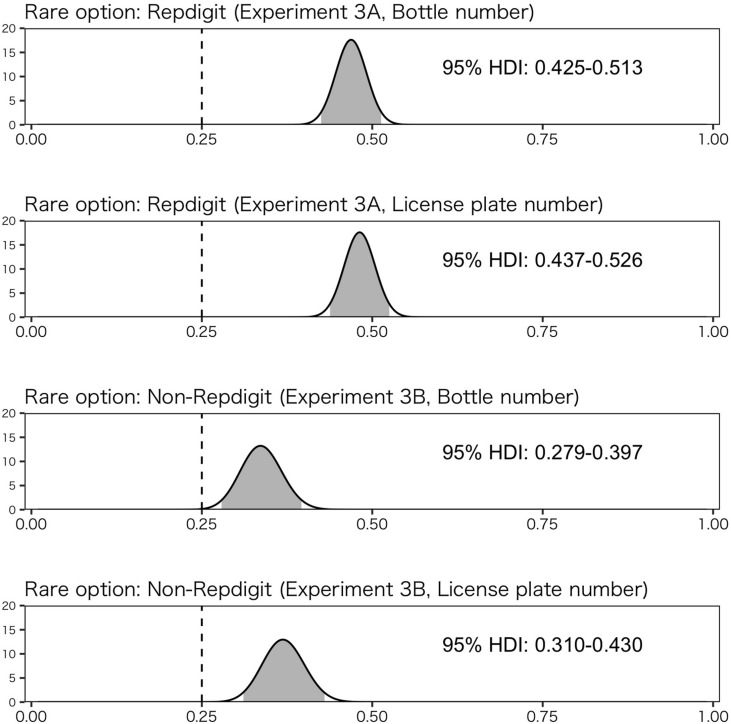
Estimation of choice rate of rare option using Bayesian method. The solid line (0.25) indicates random choice, and the gray shadow indicates the region of 95% highest-density interval (HDI).

Figure 5 shows these results. Estimated proportions of rare options in Experiment 3B were higher than 0.25 (i.e., random choice), suggesting that the rarity of the option in the context affects one’s choices. More importantly, the estimated proportion of choice rates of the rare option tends to differ between the two experiments. In particular, the estimated proportion of rare option choices tends to be higher in Experiment 3-A than that in Experiment 3-B. These results suggest that the effect of the rarity of the repdigit cannot be sufficiently explained in terms of the effect by the rarity of options in context.

In summary, rarity of option in context actually affected choices. Previous studies have indicated that option set critically affects people’s decisions. Although people tend to be loss aversive while making a decision, this can be changed by controlling option sets (Walasek and Stewart, 2015). Given that loss aversion is one of the most well-known attitudes in decision-making under risk (e.g., Kahneman and Tversky, 1979) and such an attitude can be changed by controlling option sets, it is evident that option sets play an important role in decisions and critically affect people’s decisions. Therefore, the existence of the effect of rarity in context is not so surprising. However, this effect could only explain a part of the observed results in Experiment 3-A. The comparison of estimated proportions of rare options between Experiments 3-A and 3-B indicates that the rarity of repdigit actually affects choices.

We also note that “rarity of repdigit” is directly related to “rarity of option in the context” in the real world. In the real-world cases, such as purchasing wine or getting license plate, repdigits should become “rare option” more often than non-repdigits. Thus, the experimental setting in Experiment 3-B may be peculiar in terms of real-world cases.

## Experiment 4

Our rarity hypothesis is as follows: People pay attention to the dichotomization between repdigits and non-repdigits and perceive a “rarity” in repdigits (i.e., they accurately discriminate the two classes in terms of actual frequency of possible numerical arrays). Furthermore, such feelings will affect their judgments and choices. In the precedent four experiments, results generally supported our hypothesis, especially the effects of repdigits on judgments and choices. However, the four experiments did not provide direct evidence that people actually perceived repdigits as a rarity. In Experiment 4, we conducted a rarity judgment task for numbers and directly examined whether people actually perceived repdigits as a rarity.

### Method

#### Participants

In total, 32 undergraduates (21 women and 11 men; *M*_age_ = 19.94, SD_age_ = 0.56) participated in this experiment and received a course credit. Given that this was the first experimental study about people’s rarity judgment about numbers and repdigits robustly affected people’s judgments in the precedent four experimental tasks, we set the medium effect of *d* = 0.5 (Cohen, 1988), and 80% power with the alpha level of 0.05. We needed around 30 participants based on this analysis.

#### Task and Materials

We conducted a rarity judgment task about numbers. In this task, participants were given the following instruction:

In your daily life, you encounter various three-digit numbers with regard to prices, test scores, amount for something, and so on. Please imagine such numbers. You are presented with a pair of three-digit numbers. Which number do you think you will rarely encounter in your daily life?

Participants were asked to choose one of two numbers, which they thought was rarer among the three-digit numbers they encountered in their daily lives.

For this task, we constructed pairs of three-digit numbers with the following rule. First, we categorized 900 three-digit numbers (i.e., from 100 to 999) into 9 repdigit numbers (i.e., 111, 222, 333, 444, 555, 666, 777, 888, and 999) and the other 891 into non-repdigit numbers. Then, we made two types of pairs, repdigit pair and non-repdigit pair. The repdigit pair comprised repdigit and non-repdigit numbers (e.g., 111 and 233). We constructed 27 repdigit pairs, wherein each repdigit number was used three times and combined with a randomly selected non-repdigit number. As to non-repdigit pair, two randomly selected non-repdigit numbers were combined, and 53 pairs were constructed. That is, participants made judgments for 80 pairs in total. While making pairs, 133 [106 (53 non-repdigit pairs) + 27 (repdigit pairs)] different non-repdigit numbers were used. Since we randomly selected these numbers for each participant, the presented non-repdigit numbers differed for each participant.

#### Procedure

Participants were tested individually using a computer. The participant pressed the key on the keyboard assigned for “Next,” and fixation points (asterisk) were presented for 2,000 ms on the computer screen wherein three-digit numbers were presented, followed by numbers. Participants responded by pressing one of the two keys on the keyboard that were assigned for the choice. The time that elapsed between the presentation of the numbers and the participant’s keypress was recorded. While choosing a number, participants were encouraged to respond as quickly as possible. When one of the two keys was pressed, the response was recorded and the pair of numbers disappeared. Pressing the key on the keyboard assigned for “Next” initiated the next trial (i.e., next number pair). This procedure was repeated for all number pairs. The order of presentation (i.e., in which trial repdigit pair was presented) was randomized for each participant.

### Results and Discussion

Before reporting results, we shall summarize our predictions about choice and response time. As to the choice, we predicted that participants would significantly choose repdigit number as rare number. As to the response time, if people actually feel rarity in terms of the difference between repdigit and non-repdigit, it may be easy for them to make rarity judgment in the repdigit pair since they can rely on such a difference in making judgments. In contrast, since criterion of the rarity may be vague for non-repdigit pairs, participants may find it difficult to make rarity judgments. Thus, we predicted that participants would respond to the repdigit pairs faster than non-repdigit pairs.

**FIGURE 6:**
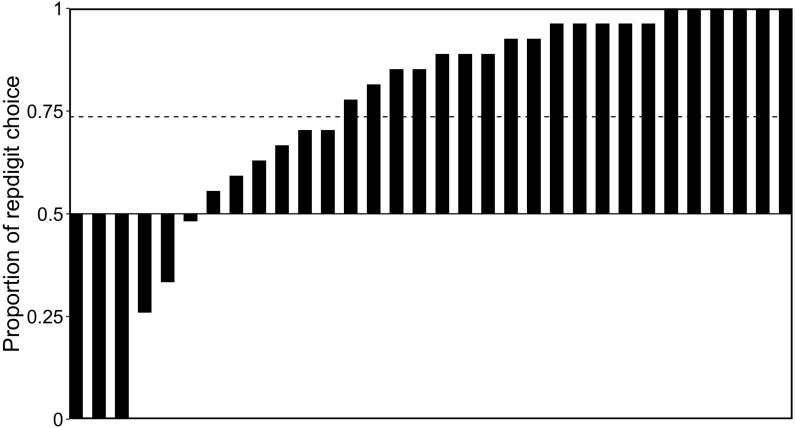
Proportions of repdigit choice in the repdigit pairs. Each bar represents the proportion of repdigit choice for each participant. The dotted bar indicates the mean proportion.

Figure 6 shows the proportions of repdigit choice in 27 repdigit pairs for each participant. We statistically examined whether the choice rate of repdigit number was higher than chance level (0.5) and found that participants significantly chose repdigit numbers as rare numbers [*M* = 0.736, *t*(31) = 4.291, *p* = 0.0002, *d* = 0.76], corroborating our prediction.

**FIGURE 7:**
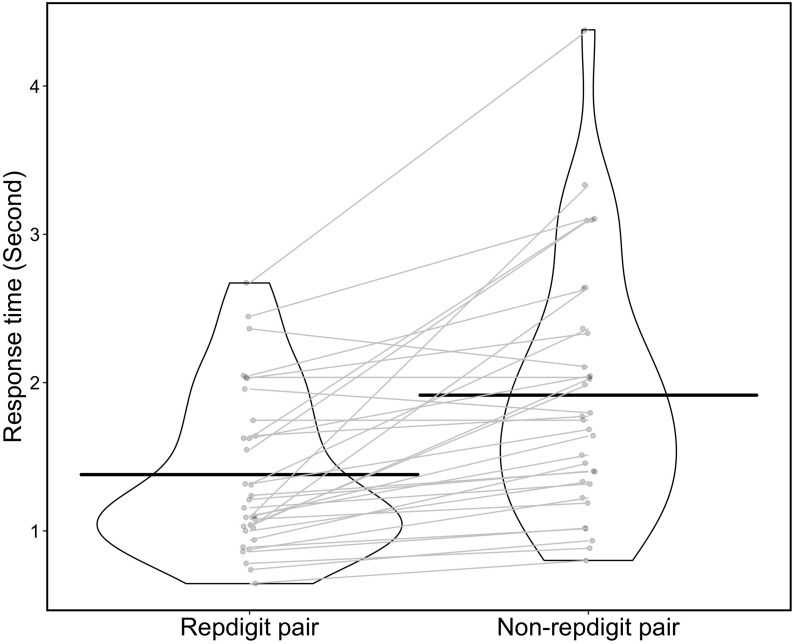
Distribution of response time (violin plot). Each point in the violin plot indicates the response time for each participant. The black solid line indicates mean response time in each pair, and the gray solid line indicates the difference in response time between repdigit and non-repdigit pairs for each participant.

In the analysis of the response time, we regarded the median of response times as the response time for each pair type and participant (i.e., median of response times for 27 pairs in repdigit pairs, and that for 53 pairs in non-repdigit pairs) and used it for the analysis. Figure 7 shows the distribution of response time. We found that the response time for the repdigit pairs was significantly faster than that for non-repdigit pairs [*M*_repdigit pairs_ = 1.380, *M*_Non–repdigit pairs_ = 1.915, *t*(31) = 5.00, *p* < 0.0001, *d* = 0.88]. Thus, our prediction was supported.

Altogether, we provided evidence that participants actually perceived repdigit numbers to be rare from choice patterns and response time, which were consistent with our predictions.

## General Discussion

We conducted five behavioral experiments to examine our hypotheses on effects of repdigits on judgments and choices. Our findings generally supported our rarity hypothesis.

Previous studies have discussed the effects of special numerical arrays, such as round numbers, on psychological processes. The effects of repdigits are essentially different from those of round numbers, which are explained mainly in terms of reference points. Since round numbers become reference points, such reference points can serve as achievement goals. People are motivated by achievement goals (e.g., Rawsthorne and Elliot, 1999) and show highly unique performances around reference points of round numbers (e.g., Pope and Simonsohn, 2011; Allen et al., 2017). However, achievement goals were not related to the present study’s choice of birthday (Experiment 1), game alternative (Experiment 2), or the wine bottle and license plate (Experiments 3-A and 3-B). Thus, effects of repdigits can be regarded as a new type of effect stemming from numerical arrays.

Kabátek and Ribar (2018) show the relationship between incidence of weddings and the “repdigit birthday” (e.g., 9.9.99) in the real world. However, to the best of our knowledge, no previous studies have examined the effect of repdigits on judgments and choices in a controlled setting. The present study is the first to examine the effects of repdigit via experimental methodology, and we provide the experimental evidence that repdigits actually affect one’s judgments and choices. Furthermore, we have also clarified the boundary as to when the repdigits affect our judgments and choices.

We believe that, in addition to round numbers and repdigits, there are some numerical arrays that may affect our judgments or choices. For example, numerical arrays with regularity – such as “123,” “369,” or “975” – will be relatively easy to memorize since they have easily detectable patterns. Then, as facilitated by the ease of pattern detection, people may find such numerical arrays special or valuable. Round numbers or repdigits are a kind of numerical array with regularity and, thus, may be easy to detect. That is, numerical arrays with regularity will be easy to detect in general. According to these facts, the ease of detection will be the primacy in assessing the distinctiveness of “special” numerical arrays from those considered to be “common.” However, we note that the psychological processes of effects by the special numerical arrays will differ depending on the features of numerical arrays. As we noted above, the effects of round numbers can be explained in terms of reference points. In contrast, we demonstrate in the present study that the effect of repdigits can be explained in terms of their rarity.

Based on these considerations, further research is necessary in order to examine the effect of numerical arrays on judgments and choices in terms of ease of detection. For example, it is necessary to examine how the difference in the ease of detecting numbers is related to the effect of numerical arrays. From this perspective, the difference between the effects by repdigits on choices and judgments, and those by numerical arrays with regularity (e.g., 123 or 246), will be understood in a more detailed manner. As a result, it is hoped that the psychological mechanisms by the effect of repdigits on one’s judgments and choices will be further clarified.

In making probabilistic or statistical judgments, people tend to think heuristically (e.g., Kahneman et al., 1982). Since repdigits are rare in the whole of numerical arrays, people may think heuristically while looking at repdigits. For example, people may infer that repdigit birthdays may be unlikely as they are rare. Accordingly, for the “real” target as in Experiment 1, people may underestimate the likelihood of repdigit birthdays. Likewise, when people are asked about the likelihood of winning the gamble as in Experiment 2, they may underestimate the likelihood of winning it for repdigit alternatives. In Experiment 1, the proportions concerning the choice of a real target in the two questions were below the chance level (0.460); this suggests the possibility that participants thought heuristically. However, the present findings do not support this possibility since we do not find statistical evidence of underestimation of the likelihood (i.e., participants’ choice patterns did not significantly deviate from random choice patterns). Rather, present findings suggest that people accurately discriminate between two classes (i.e., repdigits and non-repdigits) in terms of actual frequency of numerical arrays. As a result, repdigits affect judgments or choices only in contexts where people want to assign special meanings to numbers (i.e., ease of memorizing numbers or assigning special values for numbers). Therefore, repdigits do not always affect psychological processes.

Here, we discuss another possibility for explaining the present findings. Repdigits are structured in a simple way. Given that human cognition tends to prefer simplicity (e.g., Chater and Vitányi, 2003), the present participants may have preferred repdigits since repdigits were “simple.” Falk and Konold (1997) suggested that number strings were perceived in terms of simplicity (in their term, complexity) and subjective experience of randomness for number strings were affected by their simplicity. This indicates that people’s perception of numbers is critically affected by the simplicity of number strings. Thus, the present findings may be also explained in terms of simplicity of numbers. Although this “Preference of Simplistic Structure” hypothesis (hereafter, PSS hypothesis) may explain some present findings, we note that the PSS hypothesis cannot provide unified explanations with the following two points. First, the PSS hypothesis does not provide predictions about when repdigits are preferred. In Experiments 1 and 2, we showed that repdigits were not always preferred, suggesting that people regard repdigits and non-repdigits as the same category in some contexts. Although rarity hypothesis can predict when repdigits and non-repdigits are treated as the same, the PSS hypothesis does not specify this. Second, results in Experiment 3-B were basically inconsistent with the PSS hypothesis. The PSS hypothesis predicts that people prefer repdigit alternatives. In contrast to this prediction, results showed that the non-repdigit alternative was preferred compared to the other repdigit alternatives. Some researchers may suspect that this is also inconsistent with the rarity hypothesis. As we discussed, although the findings in Experiment 3-B were explained in part by the effect of rarity in context (i.e., the non-repdigit option was rare compared to repdigit options), this explanation was not enough for explaining the effects observed in Experiment 3-A, indicating that the rarity of repdigits actually affects our judgments and choices.

In summary, the present findings are better explained in terms of the rarity hypothesis compared to PSS hypothesis. However, we note that simplicity of numbers actually affects our perceptions of numbers and that repdigits are actually simple numerical arrays. We believe that the rarity hypothesis provides more specific explanations about why and when people prefer simple numerical strings such as repdigits.

## Conclusion

We found a new effect of numerical arrays on our judgments and choices. In addition to the previous findings on effects of numerical arrays, the present findings will make substantial contributions toward understanding psychological processes relating to people’s feelings about numerical arrays and how these affect their judgments and choices.

## Data Availability Statement

The datasets presented in this study can be found in online repositories. The names of the repository/repositories and accession number(s) can be found below: https://osf.io/fd5z6/.

## Ethics Statement

The studies involving human participants were reviewed and approved by Ethics Committee of Graduate School of Arts and Sciences at the University of Tokyo. The patients/participants provided their written informed consent to participate in this study.

## Author Contributions

All authors contributed to the experiment concept and design, and approved the final version of the manuscript for submission. SM led the data collection for Experiments 1 and 2. HH led the data collection for Experiments 3-A, 3-B, and 4, performed the data analyses, and drafted the manuscript. SM and KU provided the critical revisions.

## Conflict of Interest

The authors declare that the research was conducted in the absence of any commercial or financial relationships that could be construed as a potential conflict of interest.

## Footnotes

1 The order of presentation of alternatives was randomized for each participant in web-based experiments (Experiments 1, 2, 3-A, and 3-B).
